# Limitations of Standard Accessible Captioning of Sounds and Music for Deaf and Hard of Hearing People: An EEG Study

**DOI:** 10.3389/fnint.2020.00001

**Published:** 2020-02-18

**Authors:** Pablo Revuelta, Tomás Ortiz, María J. Lucía, Belén Ruiz, José Manuel Sánchez-Pena

**Affiliations:** ^1^Department of Computer Science, Oviedo University, Oviedo, Spain; ^2^Department of Psychiatric, Complutense University of Madrid, Madrid, Spain; ^3^Spanish Center for Captioning and Audiodescription, Carlos III University of Madrid, Leganés, Spain; ^4^Department of Computer Science, Carlos III University of Madrid, Leganés, Spain

**Keywords:** emotion, hearing impairment, audiovisual, EEG, captions, ERP

## Abstract

Captioning is the process of transcribing speech and acoustical information into text to help deaf and hard of hearing people accessing to the auditory track of audiovisual media. In addition to the verbal transcription, it includes information such as sound effects, speaker identification, or music tagging. However, it just takes into account a limited spectrum of the whole acoustic information available in the soundtrack, and hence, an important amount of emotional information is lost when attending just to the normative compliant captions. In this article, it is shown, by means of behavioral and EEG measurements, how emotional information related to sounds and music used by the creator in the audiovisual work is perceived differently by normal hearing group and hearing disabled group when applying standard captioning. Audio and captions activate similar processing areas, respectively, in each group, although not with the same intensity. Moreover, captions require higher activation of voluntary attentional circuits, as well as language-related areas. Captions transcribing musical information increase attentional activity, instead of emotional processing.

## Introduction

It is widely accepted that music produces emotional responses, being one of its defining features (Gabrielsson, 2001). Indeed, there is increasing scientific evidence on the consistency of emotional responses across listeners to the same musical features (Vieillard et al., 2008) and on the immediacy (less than 1 s) of the emotional response (Paquette et al., 2013).

However, in Spain around 5% of the population over 6 years-old presents some degree of hearing loss, according to official national statistics (INE, 2008). This means around 2.25 M people who encounter limitations when accessing audiovisual soundtrack content through television, cinema, or Internet, among other information channels.

In order to help hard of hearing people to benefit from the rights established in the UN Convention on the Rights of Persons with Disabilities (UN, 2006) concerning access to television programs, films, theater, and other cultural activities, focus was placed on captioning. Captioning is the reference assistive tool for hearing impairment, and special regulations were issued to guarantee its application [in Spain, the General Law of Audiovisual Communication (BOE, 2010) requires captioning for at least 90% of all public television broadcasts)], and its quality, considering factors as visual aspects, synchronism, presentation speed, speaker identification, or accuracy (AENOR, 2012).

Within the benefits of captioning, no significant differences were found up to date in immersion, transportation, presence, or enjoyment when watching audiovisual oeuvres either dubbed or captioned (for example, see D’Ydewalle and Van Rensbergen, 1989; Kim and Biocca, 1997; Green and Brock, 2000; Rheinberg et al., 2003; Wissmath et al., 2009). This seems to be related with automated text processing involved when reading captions, as shown in D’Ydewalle et al. (1991), D’Ydewalle and De Bruycker (2007) and Perego et al. (2015).

However, captioning offers some shortcomings. Pre-lingual deafness is associated with lower language skills and reading ability, and thus with lower caption understanding, but precisely pre-lingual profoundly deaf participants depend on alternative methods of information assimilation as captions (Gulliver and Ghinea, 2003). When captions are added, attention is drawn to captions, resulting in a reduction in the level of video information assimilated, though captions provide a greater level of context (Gulliver and Ghinea, 2003) or improve the comprehension when added to sign language interpreter videos (Debevc et al., 2015).

Another issue is that when dealing with non-verbal sounds, national regulations establish verbal cues: sound effects and music must be subtitled in the upper right of the screen formatted in brackets, e.g., (Applause), (Phone). In the case of music, information of the type of music, sensation transmitted, and identification of the piece (title, author) must be included, e.g., (Rock music), (Horror music), (Adagio, Albinoni). This verbal representation does not include the emotional information of sounds and music (Pehrs et al., 2014). Although many experiments were conducted with captions, none of them deals with the musical representation of non-verbal information.

Our main hypothesis is that captions cannot elicit the same emotional and behavioral reactions than sound or music. Moreover, captions would produce “lower emotional effects” than the auditory correlates, as they require conscious and selective attention (Gulliver and Ghinea, 2003).

Among the many ways these limitations can be measured and quantified, in this study, we chose event-related potential (ERP) measurements before emotional motor response by means of EEG. The focus of the present study is to examine what happens just before a motor response (associated to emotion detection) while watching videos with audio or captions in two groups of participants: normal hearing and deaf or c subjects.

The decision of using the ERPs prior to motor response is based on the following results: the emotional and cognitive networks involved in decision making can be tracked by ERPs (Olofsson et al., 2008; Imbir et al., 2015). There are negative ERPs close to motor response present in anticipatory processes that reflect the emotional and cognitive processing of stimuli, such as Readiness Potential (Pedersen et al., 1998), Movement Preceding Negativity (Brunia and van Boxtel, 2001), Negative Shift Potential (Ortiz et al., 1993; Duncan et al., 2009), or Decision Preceding Negativity (DPN; Bianchin and Angrili, 2011). DPN is the last salient slow negative potential before a willed risky decision (Bianchin and Angrili, 2011) associated with emotional processes. Before motor response, researchers have found a negative wave around 150 ms that is associated with neurophysiological processes related to decision making (Shibasaki et al., 1980; Ortiz et al., 1993).

## Materials and Methods

### Participants

Two groups of participants were recruited.

In one group, 16 participants with self-reported normal hearing were recruited, eight females and eight males, aged between 20 and 60 (mean: 39.83, *SD*: 12.24), 24.1% with a high school degree, 27.6% with a college degree, and 48.3% with post-graduate studies.

In the other group, 13 participants with self-reported hearing loss were recruited, seven females and six males, aged between 20 and 60 (mean: 39.4, *SD*: 12.21), 38.5% with a high school degree, 38.5% with a college degree, and 23.1% with post-graduate studies.

The self-reported hearing losses were classified according to the Audiometric Classification of Hearing Impairments of the International Bureau for Audiophonology (BIAP, 1996): mild hearing loss (between 20 and 40 dB), moderate hearing loss (between 41 and 70 dB, speech is perceived if the voice is loud, and the subject understands better what is being said if he can see his/her interlocutor), severe hearing loss (between 71 and 90 dB, speech is perceived if the voice is loud and close to the ear, loud noises are perceived), very severe hearing loss (between 91 and 119 dB, speech is not perceived, only very loud noises are perceived), and total hearing loss (over 120 dB).

Four participants had moderate hearing loss and used hearing aids, four participants had severe loss, used hearing aids and three of them had cochlear implant, and one participant had total loss and had cochlear implant.

All of them signed an informed consent approved by the Bioethical Committee of the Carlos III University of Madrid and filled out a survey concerning demographic information, level of studies, and degree of hearing loss.

### Materials

#### Stimuli

The visual stimuli used were extracted from the “Samsara” documentary in order to select neutral sequences without story or associated dramaturgy. The “Samsara”’ documentary^1^ is composed of sequences of soft images of nature and human society from 25 countries with musical background but without dialog or written messages. Forty video extracts of 10-s length were selected based on the absence of plane changes during 10 s. The original soundtrack was removed, and a 2-s fade-in and fade-out were applied to soften the transitions. An auditory stimulus was added to each fragment. These stimuli proceeded from an audio database: the fragments and the instant in which they appeared were assigned randomly (between seconds 2 and 8 to avoid the fades). A caption corresponding to these auditory stimuli was added to each muted fragment. The captions were generated by a specialist at the Spanish Center for Captioning and Audio description (CESyA) following the Spanish regulation (AENOR, 2012).

A final video was built combining the 40 audio fragments and the corresponding 40 captioned fragments. These 80 fragments were randomly sorted, and the final video was split into five sets, allowing 20 s of rest between each set.

#### Hardware

In the presented experiment, two computers were involved, one triggering the video and sending temporal marks to the EEG amplifier to locate the stimuli, with screen and speakers pointing to the participant, and another one registering the EEG data. This last one allows a high-density (128 channel) EEG recordings, obtained using a custom-designed electrode Neuroscan cap and an ATI EEG system (Advantek SRL). Impedances were kept under 5 kΩ. Additional channels were included to monitor eye movement (right and left lateral canthi and superior and inferior orbits of the left eye). The reference electrodes were placed on the mastoids, and the ground electrode was placed on the forehead. Data were processed to an average reference following acquisition with a band-pass filter of 0.05–30 Hz and a sample rate of 512 Hz. An artifact rejection criterion of 100 μV was used to exclude eye blinks. Individual subject averages were visually inspected to insure that clean recordings were obtained. Eye and muscle movement artifacts were identified off-line on a trial-by-trial basis through visual inspection, and they were removed prior to data averaging and ERP analysis. Noisy channels were sparingly replaced with linear interpolations from clean channels (around 6 ± 3.5 channels per record and subject). From the remaining artifact-free trials, averages were computed for each participant and each condition. The analysis epochs for ERPs were 500 ms before motor response. EEG analysis was carried out on frequent (non-target) trials to avoid contamination by motor-related neural activity associated with making a response. ERPs obtained were averaged separately for each condition and each subject. A Bayesian Model Averaging (BMA) analysis over all electrodes was performed by opening a time window of −20 to +20 ms around the highest negative amplitude peak measured in Cz electrode.

### Procedure

Participants were cited in individual sessions. They were first asked to fill in a survey including questions about their age, gender, education level, type, and degree of hearing loss and hearing aids if applicable.

Then, they were asked to sit in an armchair facing a 17″ screen with speakers placed at 1.5 m in front of the participant. They were asked to remove their hearing aids, but to keep their glasses on if needed. The 128-EEG cap was fixed to their head. A press button was placed under their left hand. Participants were explained that they were going to watch a video and were asked to press the button grabbed in their left hand whenever they felt any emotion while watching the video. Lights in the room were turned off, and the corresponding video was launched. Normal hearing participants watched the video with soundtrack (audio and captioned sequences), while participants with hearing loss watched the video without soundtrack (muted and captioned sequences). The press button in their left hand was connected with one of the computers, and each pressure was transmitted and registered as a mark in the EEG track.

## Results

### Behavioral Response

We registered the total number of times each participant pressed the button with their left hand indicating they were feeling an emotion. The scores were registered for three conditions: Audio (pressures occurring during audio fragments display), Caption (pressures occurring during captioned fragments display), and Mute (pressures occurring during muted fragment display).

Nonparametric Mann–Whitney tests were used to compare the number of button presses in the different conditions and groups. The Mann–Whitney statistic was selected as the Shapiro–Wilk test rejected normality in some conditions, and the size of the samples was not sufficiently large (less than 20) to assume the normal distribution in the rest of the conditions. The results are shown in Table 1.

**Table 1:** Comparisons of number of button presses for each group in each condition and between groups for Caption condition.

Hearing	Hearing loss		
Audio	Mute		
19.87 ± 9.63	7 ± 4.1		
Caption	Caption		
9.81 ± 9.69	7.15 ± 4.86	*P*-value**	0.79486
*P*-value*	*P*-value*		
0.00528	0.52218		
<0.05	>0.05		

*P-values of standardized Mann–Whitney statistics comparing Audio/Caption conditions in Hearing group and Mute/Caption conditions in Loss Hearing Group. **P-values of standardized Mann–Whitney statistics for Caption Condition in Hearing and Hearing Loss Group.

A Mann–Whitney test was conducted to compare the number of button presses in Caption and Audio conditions in the normal hearing group. The standardized results show a significant difference (*p* = 0.00528) in the scores for Audio (19.87 ± 9.63) and Caption (9.81 ± 9.69) conditions, suggesting that video auditory stimuli produced a number of emotional reactions twice as much as the captions stimuli did. A second Mann–Whitney test was conducted to compare the number of button presses in Caption and Mute conditions in the hearing loss group. There was no significant difference (*p* = 0.52218) in the scores for the Mute (7 ± 4.1) and Caption (7.15 ± 4.86) conditions, suggesting that captions do not produce additional emotional reactions to the visual stimuli.

Finally, a Mann–Whitney test was conducted to compare the number of button presses in Caption condition in the normal hearing group and in the hearing loss group. There was no significant difference (*p* = 0.79486) in the scores for the hearing group (9.81 ± 9.69) and the hearing loss group (7.15 ± 4.86), suggesting that the emotional reaction to captions is similar in both groups.

### ERP Waves Before Emotional Response Onset

**Figure 1:**
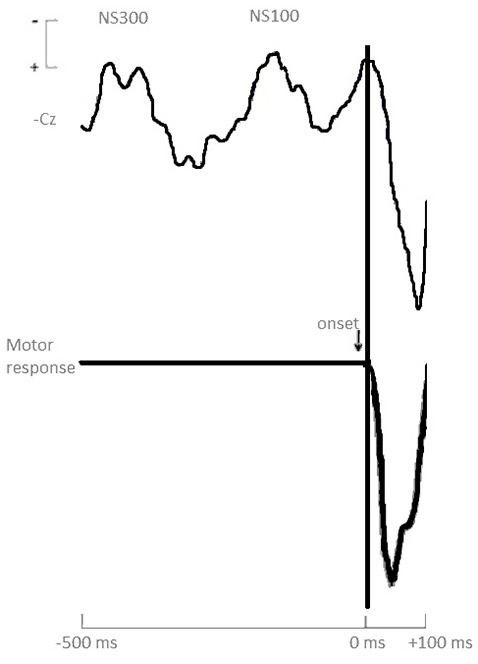
Grand averages of stimulus-synchronized cerebral waveforms (Cz) prior to motor response onset. Calibration signal at the left indicates ±10 μV for the cerebral responses.

Figure 1 shows the response onset-synchronized cerebral responses recorded from the vertex Cz electrode for the task. Prior to the emotional response (button press), two negative waves were found: an early negative shift around 300 ms (labeled NS300 from now on) and a closely negative wave around 100 ms (labeled NS100) prior to onset emotional response (button press).

### Source Localization

NS300 and NS100 show significantly greater activation in both groups in temporal middle and inferior lobe for all video fragments.

**Figure 2:**
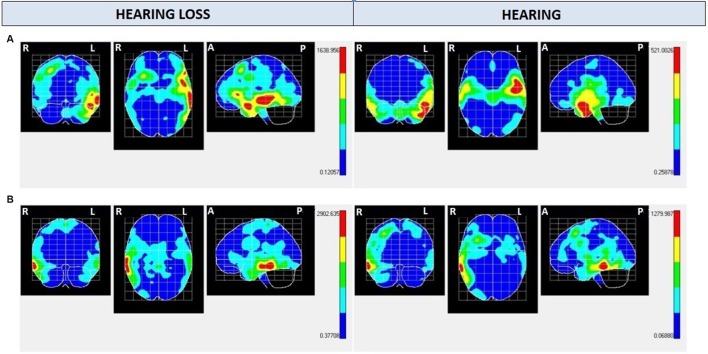
Negative shift around 300 ms (NS300) mean electrical maps with **(A)** Mute/Audio condition and **(B)** Caption condition in each group. SPMs were computed based on a voxel-by-voxel Hotelling T^2^ test against zero. Maximal intensity projection areas are displayed in yellow/red color. Averaging [Bayesian Model Averaging (BMA)] analysis was made by opening a time window of −20 to +20 ms starting from the highest negative amplitude peak measured in Cz electrode.

Regarding NS300 maps (Figure 2 and Tables s 2, 3), NS300 (with an average amplitude of −1.8 μV and *SD* of 0.67) shows high activation in the left temporal pole (TP) lobe for Audio and Mute conditions in the hearing group and the hearing loss group, respectively. The difference between both groups concerns the magnitude obtained with the Hotelling’s T^2^ test that shows (given an equal number of samples in all maps) greater activation of these cerebral areas in the hearing loss group (>1,400) compared with the hearing group (≅500). In addition, high activation appears in the frontal inferior lobe only in the hearing loss group. For the Caption condition, activation is found in the right temporal lobe in both groups, with greater activation in the hearing loss group (>2,500) compared with the hearing group (≅1,200).

**Table 2:** Hearing loss group NS300/NS100 wave summary.

	AAL	BA	*X*	*Y*	*Z*	Activation (T^2^)
**NS100 wave**
Mute	Temporal Mid R	21	65	−39	−16	917.45
	Temporal Inf R	20	66	−35	−20	736.67
Caption	Temporal Mid R	21	66	−22	−13	828.67
	Temporal Inf R	20	54	−7	−31	726.78
	Temporal Pole Mid R	20	46	8	−23	573.28
**NS300 wave**						
Mute	Temporal Mid L	21	−66	−31	−8	1,624.87
	Temporal inf L	20	−66	−31	−14	1,484.78
	Temporal Pole Sup L	38	−46	10	−21	1,434.96
	Frontal Inf Tri L	45	−54	28	4	1,347.67
Caption	Temporal Inf R	20	66	−38	−12	2,902.56
	Temporal Mid R	21	66	−22	−13	2,579.89

*Note. NS300/NS100 wave summary of maximal intensity projection areas statistically significant based on a voxel-by-voxel Hotelling T^2^ test against zero (*p* < 0.001), with each specific brain area localization. Abbreviations: AAL, Automated Anatomical Labeling corresponding to Probabilistic Brain Atlas; BA, Brodmann areas; X, Y, Z, coordinates in three spatial axes according to the MNI coordinate system of the maximum point T^2^ in the anatomical structure*.

**Table 3:** Hearing group NS300/NS100 waves summary.

	AAL	BA	*X*	*Y*	*Z*	Activation (T^2^)
**NS100 wave**
Audio	Parietal Sup R	7	22	−62	64	1,304.19
	Temporal Mid R	21	66	−23	−13	549.56
	Temporal Inf R	20	50	−2	−36	437.68
Caption	Temporal Mid R	21	66	−23	−13	1,808.11
	Temporal Inf R	20	66	−38	−12	1,843.91
**NS300 wave**
Audio	Temporal Pole Sup L	38	−12	6	−20	525.79
	Temporal Inf L	20	−50	−2	−36	513.56
	Temporal Mid L	21	−50	5	−23	506.34
Caption	Temporal Mid R	21	66	−35	−13	1,265.89

*Note. NS300/NS100 wave summary of maximal intensity projection areas statistically significant based on a voxel-by-voxel Hotelling T^2^ test against zero (*p* < 0.001), with each specific brain area localization*.

**Figure 3:**
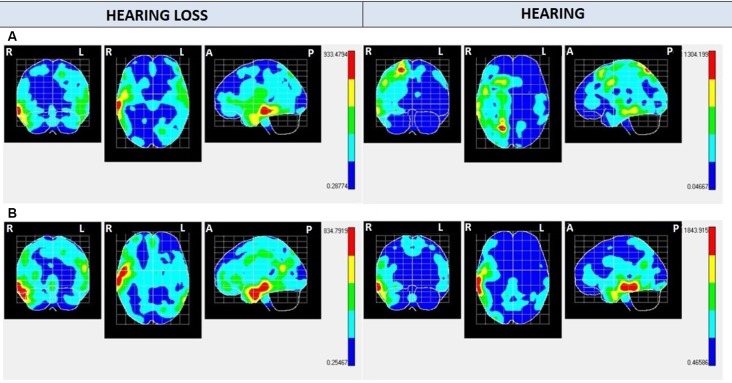
NS100 mean electrical maps with **(A)** Mute/Audio condition and **(B)** Caption condition in each group. SPMs were computed based on a voxel-by-voxel Hotelling T^2^ test against zero. Maximal intensity projection areas are displayed in yellow/red color. Averaging (BMA) analysis was made by opening a time window of −20 to +20 ms starting from the highest negative amplitude peak measured in Cz electrode.

Regarding NS100 maps (Figure 3 and Tables s 2, 3), activation is found in the right temporal lobe in both groups for Audio, Mute, and Caption conditions. For Audio and Mute conditions, NS100 (average amplitude of −2.4 μV and *SD* of 1.27) shows a significantly greater activation in the hearing loss group. Activation in the right parietal lobe for the Audio condition appears only in the hearing group. For the caption stimuli, greater activation is found in the hearing group (≅1,800) compared with the hearing loss group (≅800).

### NS Latencies

In the hearing group, the average NS100 latency measured was 165.85 ms (±6.14) for the Audio condition and 161.91 ms (±8.22) for the Caption condition. In the case of NS300, the results were 377.79 ms (±50.57) for Audio and 370.08 ms (±43.99) for Caption. No significant differences were found in the NS100 or NS300 latencies between the Audio and Caption conditions in this group.

In the hearing loss group, the average NS100 wave latency for the Mute condition was 116.15 ms (±19.30) and 125.84 ms (±18.17) for the Caption condition. Regarding the NS300 wave, the latency was 338.46 ms (±7.77) for the Mute and 334.15 ms (±6.15) for the Caption. No significant differences were found in NS100 or NS300 latencies between the Mute and Caption conditions in this group.

Significant differences between groups where found in the Caption condition. Table 4 shows the Mann–Whitney test results comparing NS100 and NS300 latencies in the Caption condition between hearing and hearing loss groups. For NS100, a significant difference (*p* = 0.0001) was found between the hearing group (165.85 ± 6.14) and the hearing loss group (125.85 ± 18.17). For NS300, a significant difference (*p* = 0.00078) was likewise found between the hearing group (377.79 ± 50.57) and the hearing loss group (334.15 ± 6.15). These results suggest that the reaction time is lower in the hearing loss group for the Caption condition.

**Table 4:** Comparisons of NS100 and NS300 latencies between groups for Caption condition.

NS100		NS300
Hearing	165.85 ± 6.14	377.79 ± 50.57
Hearing loss	125.85 ± 18.17	334.15 ± 6.15
*P*-value	0.0001	0.00078
	<0.05	<0.05

*P-values of standardized Mann–Whitney statistics*.

## Discussion

An important result from our study is the existence of two differentiated negative ERPs, labeled NS100 and NS300, regarding the latency and cerebral activation produced prior to the motor response (button press), without significant differences between both groups. The negative ERPs close to the motor response present in anticipatory processes reflect the emotional and cognitive processing of stimuli and neurophysiological processes related to decision making (Shibasaki et al., 1980; Ortiz et al., 1993; Duncan et al., 2009; Bianchin and Angrili, 2011). Different authors state that the negative component immediately previous to the motor response (NS100 in our case) is related to cognitive processes needed to activate the motor programs associated with the decision making and to the efficiency of the executive behavior (Bianchin and Angrili, 2011). These results indicate a clear cognitive and emotional processing in both groups prior to the motor response.

Comparing the hearing and hearing loss groups’ brain activation with Audio and Mute conditions, respectively, it was found that both groups activate the same left temporary (lower, middle, and pole) areas, meaning that images activate the same areas, and probably the same emotional and cognitive processes. The lower and middle temporal areas are associated with visual and auditory processing, object and face recognition, and word meaning (Pehrs et al., 2017). The TP is part of the association cortex and is involved in multimodal sensory integration (Olson et al., 2007; Skipper et al., 2011), and it has been implicated in various higher-order functions of socioemotional cognition and empathic behavior (Altmann et al., 2012; Aust et al., 2013; Carlson et al., 2014; Parkinson and Wheatley, 2014). To test whether TP acts as a semantic hub integrating over complex social cues, naturalistic stimuli were employed: empathy-evoking movie sequences depicting protagonists undergoing emotional experiences. Sharing such experiences with filmed protagonists requires a continuous neural multisensory integration of visual, auditory, and contextual information (Raz et al., 2014).

The difference appreciated between the normal hearing and hearing loss groups with Audio and Mute conditions, respectively, regarding the NS300 maps, was the greater activation of these cerebral areas in the hearing loss group. Numerous studies justified that the increase in the amplitude of the evoked potentials would be associated with greater cognitive effort of the task, more complex processing, and processing intensity (Moreno et al., 2016; Romero-Rivas et al., 2016; Sanchez-Lopez et al., 2016).

In addition, the hearing loss group showed activation in the left frontal inferior pole in Mute condition. This NS300 frontal activity could be associated with higher voluntary emotional attentional resources and integration of cognitive and emotional processes when perceiving external stimuli (Böcker et al., 2001; De Marino et al., 2006).

Regarding Caption condition, comparing the hearing and hearing loss group, we found that both groups activated the right temporal inferior and medium areas (activity is centered in visual processing and word recognition), but again, the hearing loss group activated these areas with higher intensity. The shorter reaction time in the hearing loss group can be related to this higher activation.

As for the laterality of the processes, we found highest left hemisphere activity for NS300 in both groups for Audio and Mute conditions, which seems to be related to attentional positive emotional processes. Other scientific studies demonstrated that the valence of emotions is represented bilaterally in our brain, emerging differentially positive in the left hemisphere and negative in the right hemisphere (Silberman and Weingartner, 1986). Instead, NS100 showed activation in the right hemisphere in both groups for Audio and Mute conditions.

## Conclusions

Auditory stimuli produced a significant number of emotional reactions, in addition to the emotional reactions produced by the visual components that captions did not produce. Related to EEG measures, two new EEG waves were found, labeled negative shift around 100 ms (NS100) and 300 ms (NS300) prior to emotional response onset. These waves were present with different intensities or in different areas in the normal hearing and hearing loss groups, demonstrating that:

− Both groups mobilized temporal perception and processing areas.
− The deaf group mobilized these areas with much higher intensity and adding voluntary cerebral resources (frontal areas) when watching muted videos without captions.
− Hearing people mobilized these resources with moderate intensity levels and activated perception integration areas (Parietal Sup) when watching the same videos with audio.
− The presence of captions increased and focused the activation of visual and word processing areas in both groups.

On the one hand, these results indicate that when a subject with hearing loss is watching a video without captions, a higher voluntary attentional effort is needed prior to motor response compared with the normal hearing group. According to previous works, this greater energy is related to higher brain resource consumption as a consequence of hearing loss. If we add captions to the video, this attention effort increases and focuses on visual and word processing. Other studies with deaf and hard of hearing participants showed that captions cause a shift in attention from video information to captioned information (which results in an increased level of information assimilated from captions to the detriment of the information assimilated from the video sources), especially in participants with profound and severe hearing loss who rely on captions as the principal source of context information.

On the other hand, these results show that auditory stimuli produce significantly more number of emotional reactions than captions. Studies on the emotional response to music showed that less than 2 s of music can produce basic emotional states as happiness, sadness, or fear, and that the emotional response depends on musical parameters such as mode, tempo, register, dynamics, articulation, or timbre. These parameters do not have a literal translation, and thus, captions transcribing information associated with non-verbal sounds generate additional intensity of attentional activity, instead of emotional response.

Finally, we found that, when watching captioned videos, all the cerebral activations are produced in the right hemisphere, while when they are not present, both hemispheres interact. The explanation of this fact remains open for further research since the generally assumed approach, establishing that the right hemisphere is the one in charge of processing negative emotions, and the left hemisphere positive emotions, does not bring light to these findings.

With this study, we want to contribute from a scientific basis to the enrichment of the deaf and hard of hearing people audio-visual experience. Our main conclusion is that if we want deaf people to feel the emotion produced by sounds in a similar manner as hearing people do, we need to provide other non-verbal representations of the sound, exploring other stimuli, rather than literal captions, triggering more direct emotional reactions. There is increasing research on the correspondences between the sense of hearing and the sense of touch and, thus, the potential of vibrotactile technologies to produce musical experience (Vieillard et al., 2008; Russo et al., 2012; Hopkins et al., 2016). Different devices have already been designed that apply tactile vibrations to the skin (fingertip, back, forefoot…), reproducing musical features as rhythm to enhance musical experience in deaf and hard of hearing people. Another example of different creative representation is the enriched captioning of the Russian film “Night watch”^2^, which embeds the captions in a very creative way into the scene visual composition, combining fonts, colors, animations, and other artistic resources in a radical application of the Design-for-All paradigm (Design for All Foundation, 2019). The Design-for-All paradigm is much more than an accessibility guideline. It states that designing products, thinking about the different casuistic the public may present, makes the oeuvre not only accessible for a wider group but also more consistent, homogeneous, and even more enhanced for everyone, independently of the eventual disabilities. Thus, we suggest the creative designer, art creators, and other public-related professionals to integrate this paradigm in their works.

## Data Availability Statement

The datasets generated for this study are available on request to the corresponding author.

## Ethics Statement

The studies involving human participants were reviewed and approved by Carlos III University of Madrid Ethics Committee. The patients/participants provided their written informed consent to participate in this study.

## Author Contributions

TO and PR contributed to all aspects of the work. BR and JS-P contributed to the conception and design of the study. ML contributed to the data analysis, discussion, and article writing. All authors contributed to manuscript revision, read and approved the submitted version.

## Conflict of Interest

The authors declare that the research was conducted in the absence of any commercial or financial relationships that could be construed as a potential conflict of interest.

## Footnotes

^1^https://www.filmaffinity.com/es/film269079.html

^2^https://www.filmaffinity.com/en/film670027.html
